# One-Step Detection of the 2009 Pandemic Influenza A(H1N1) Virus by the RT-SmartAmp Assay and Its Clinical Validation

**DOI:** 10.1371/journal.pone.0030236

**Published:** 2012-01-25

**Authors:** Yuki Kawai, Yasumasa Kimura, Alexander Lezhava, Hajime Kanamori, Kengo Usui, Takeshi Hanami, Takahiro Soma, Jean-Étienne Morlighem, Satomi Saga, Yuri Ishizu, Shintaro Aoki, Ryuta Endo, Atsuko Oguchi-Katayama, Yasushi Kogo, Yasumasa Mitani, Takefumi Ishidao, Chiharu Kawakami, Hideshi Kurata, Yumiko Furuya, Takayuki Saito, Norio Okazaki, Masatsugu Chikahira, Eiji Hayashi, Sei-ichi Tsuruoka, Tokumichi Toguchi, Yoshitomo Saito, Toshiaki Ban, Shinyu Izumi, Hideko Uryu, Koichiro Kudo, Yuko Sakai-Tagawa, Yoshihiro Kawaoka, Aizan Hirai, Yoshihide Hayashizaki, Toshihisa Ishikawa

**Affiliations:** 1 Omics Science Center, RIKEN Yokohama Institute, Tsurumi-ku, Yokohama, Japan; 2 Yokohama City Institute of Health, Isogo-ku, Yokohama, Japan; 3 Kanagawa Prefectural Institute of Public Health, Chigasaki, Japan; 4 Hyogo Prefectural Institute of Public Health and Consumer Sciences, Hyogo-ku, Kobe, Japan; 5 Chiba Prefectural Togane Hospital, Togane, Japan; 6 Isumi Medical Center, Isumi, Japan; 7 National Center for Global Health and Medicine, Shinjuku-ku, Tokyo, Japan; 8 Institute of Medical Science, University of Tokyo, Shirokanedai, Minato-ku, Tokyo, Japan; Centers for Disease Control and Prevention, United States of America

## Abstract

**Background:**

In 2009, a pandemic (pdm) influenza A(H1N1) virus infection quickly circulated globally resulting in about 18,000 deaths around the world. In Japan, infected patients accounted for 16% of the total population. The possibility of human-to-human transmission of highly pathogenic novel influenza viruses is becoming a fear for human health and society.

**Methodology:**

To address the clinical need for rapid diagnosis, we have developed a new method, the “RT-SmartAmp assay”, to rapidly detect the 2009 pandemic influenza A(H1N1) virus from patient swab samples. The RT-SmartAmp assay comprises both reverse transcriptase (RT) and isothermal DNA amplification reactions in one step, where RNA extraction and PCR reaction are not required. We used an exciton-controlled hybridization-sensitive fluorescent primer to specifically detect the HA segment of the 2009 pdm influenza A(H1N1) virus within 40 minutes without cross-reacting with the seasonal A(H1N1), A(H3N2), or B-type (Victoria) viruses.

**Results and Conclusions:**

We evaluated the RT-SmartAmp method in clinical research carried out in Japan during a pandemic period of October 2009 to January 2010. A total of 255 swab samples were collected from outpatients with influenza-like illness at three hospitals and eleven clinics located in the Tokyo and Chiba areas in Japan. The 2009 pdm influenza A(H1N1) virus was detected by the RT-SmartAmp assay, and the detection results were subsequently compared with data of current influenza diagnostic tests (lateral flow immuno-chromatographic tests) and viral genome sequence analysis. In conclusion, by the RT-SmartAmp assay we could detect the 2009 pdm influenza A(H1N1) virus in patients' swab samples even in early stages after the initial onset of influenza symptoms. Thus, the RT-SmartAmp assay is considered to provide a simple and practical tool to rapidly detect the 2009 pdm influenza A(H1N1) virus.

## Introduction

The 2009 pandemic (pdm) influenza A(H1N1) virus, a new strain of virus identified in Mexico in April 2009, caused outbreaks on both local and global scales with severe consequences for human health and the global economy [1]–[4]. In Japan, the first 2009 pdm A(H1N1) influenza case was reported on May 9, 2009, followed by more than 200 cases reported in the Osaka and Kobe areas by May 21, 2009 [5]. Thereafter, the pandemic infection spread widely throughout Japan, where the numbers of influenza cases reported per sentinel provider peaked at 39.63 in November 2009 with over 200 fatal cases owing to infection with the 2009 pdm influenza A(H1N1) viruses. The 2009 pdm influenza A(H1N1) viruses appear to have a high evolutionary rate [6], and mutated viruses rapidly circulated around Japan via modern traffic networks. Phylogenic analyses have revealed that the 2009 pdm influenza A(H1N1) viruses in Japan differed between the very early phase and the peak phase of the pandemic [6], [7].

The 2009 pdm influenza virus is a triple combination comprising RNA segments from both North American and Eurasian swine influenza and from avian influenza viruses [8]. Namely, the 2009 pdm influenza A(H1N1) virus possesses PB2 and PA genes of North American avian virus origin, a PB1 gene of human H3N2 virus origin, HA (H1), NP, and NS genes of classical swine virus origin, and NA (N1) and M genes of Eurasian avian-like swine virus origin [9], [10]. Unlike most avian and swine A influenza viruses that can sporadically infect humans via animal to human transmission but lack the ability to be transmitted from human to human, the 2009 pdm influenza A(H1N1) virus showed a strong ability to be transmitted from human to human through respiratory droplets. In the case of infection with the 2009 pdm influenza A(H1N1) virus, many groups of patients, including the immune-compromised and those with underlying chronic conditions such as asthma or chronic obstructive pulmonary disease, were vulnerable to complications that resulted in mortality [11], [12]. According to WHO statistics, this pdm virus has killed more than 18,000 people since it emerged in April 2009 [5], [11].

By May 2010, the pandemic began to taper off, and the number of cases declined steeply. On August 10, 2010, WHO announced the post-pandemic period [5]. There are still concerns, however, that the 2009 pdm influenza A(H1N1) virus might mutate or re-assort with existing influenza viruses giving it more virulence when it returns. In fact, the 1918 Spanish flu pdm virus was relatively mild in its first wave and acquired more virulence when it returned in the winter [13]. Furthermore, the wide-spread administration of oseltamivir might contribute to the emergence of oseltamivir-resistant 2009 pdm influenza A(H1N1) viruses as dominant variants. Owing to the heavy use of oseltamivir in Japan, the oseltamivir-resistance mutation rate (1.2%) can be expected to greatly increase in the upcoming season [14]. Thus, preparedness on a global scale against a potentially more virulent and/or drug-resistant strain is highly recommended.

Rapid influenza diagnostic tests (lateral flow immuno-chromatographic tests) were widely used to detect the influenza viral nucleoprotein antigen; however, they have a relatively low sensitivity and yield about 30% false negative results [15]. While PCR is a sensitive method, it requires laborious steps, such as RNA extraction and reverse transcriptase reaction steps. Therefore, it is crucially necessary to develop a simple, rapid, and highly sensitive method that enables clinical detection of the 2009 pdm influenza A(H1N1) virus. For this reason, we have developed a one-step method, named “RT-SmartAmp assay”, that detects the HA segment of the 2009 pdm influenza A(H1N1) virus. The SmartAmp method was previously developed as an isothermal nucleotide amplification method [16]–[18]. The RT-SmartAmp assay developed in this study combines both reverse transcriptase (RT) and isothermal DNA amplification reactions in a single step, such that the required detection time is only about 40 min, and tangled RNA extraction is not required. This paper reports the technical development and clinical validation of the RT-SmartAmp assay for rapid detection of the 2009 pdm influenza A(H1N1) virus.

## Results

### Preparation of SmartAmp primers to detect the HA segment of the 2009 pdm influenza A(H1N1) virus

**Figure 1:**
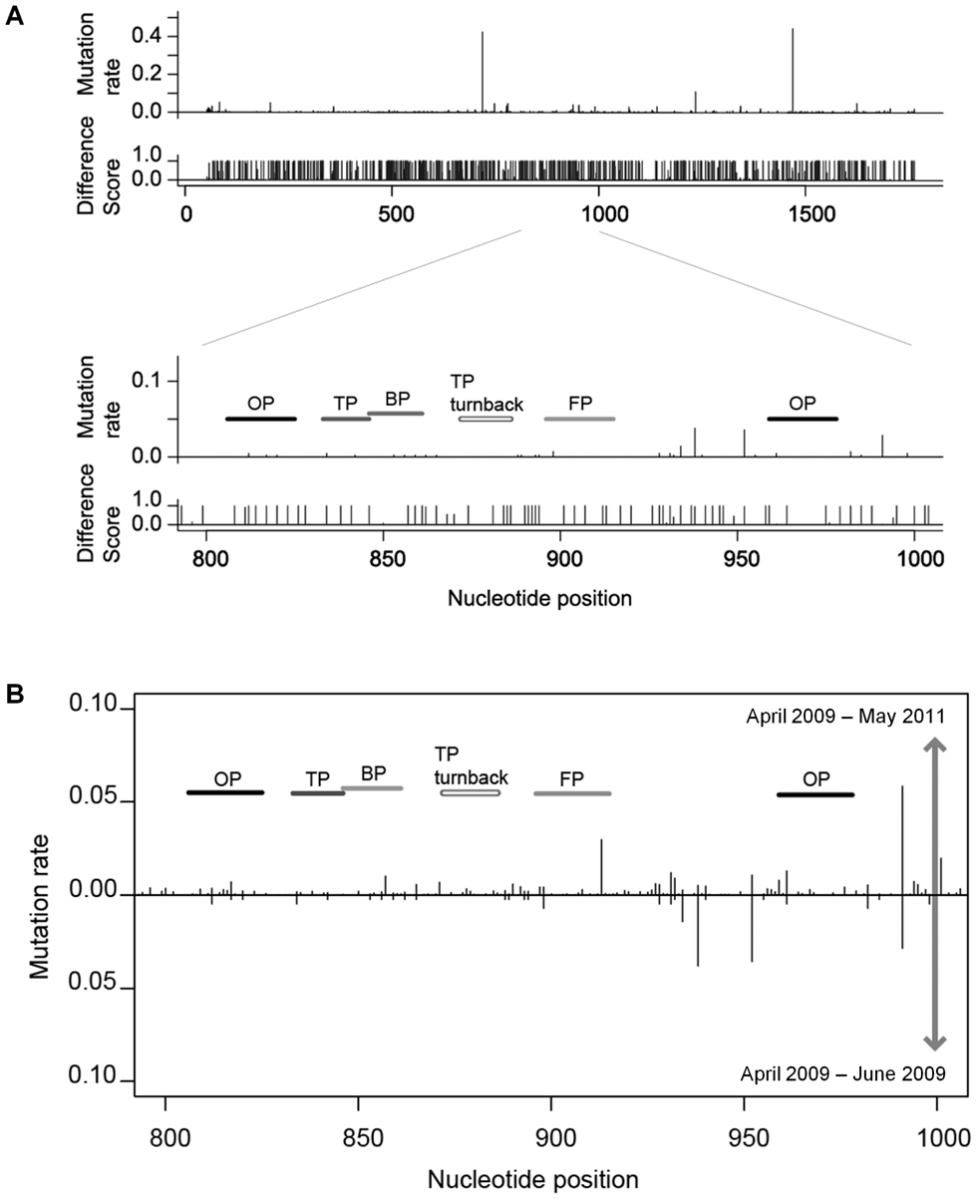
Preparation of SmartAmp primers to detect the HA segment of the 2009 pdm influenza A(H1N1) virus. A. Mutation rate and difference score in the consensus sequence of the HA segment. Nucleotide sequences of the HA segment of 2009 pdm influenza A(H1N1) viruses were obtained from the NCBI Influenza Virus Resource database and aligned by using the MUSCLE program to gain the consensus sequence of the HA segment. The mutation rate at each base position was calculated as described in Materials and Methods. The difference between 2009 pdm and seasonal A(H1N1) viruses was calculated at each position in the nucleotide sequence of the HA segments to gain the difference score. B: Comparison of data acquired in 2009 and 2011 as to the mutation rates in the HA segment of the 2009 pdm influenza A(H1N1) viruses.

Based on data from the NCBI Influenza Virus Resource database, we have analyzed the nucleotide sequences of the 2009 pdm influenza A(H1N1) virus to obtain consensus nucleotide sequences and to calculate the mutation rate at each consensus nucleotide sequence position in the HA segment of the virus. In addition, we compared these nucleotide sequences between the 2009 pdm influenza A(H1N1) virus and other seasonal A(H1N1) viruses to calculate the difference scores, as described in Materials and Methods. Figure 1 depicts both the mutation rate and the difference score at every nucleotide position in the HA segment of the 2009 pdm influenza A(H1N1) virus. By considering the criteria of (*a*) low mutation rates and (*b*) high difference scores, we selected one target sequence region (nucleotide positions 595–1048) in the HA segment (Figure 1). The sequence of this target region contained few mutations, but was specific for the 2009 pdm influenza A(H1N1) virus.

**Figure 2:**
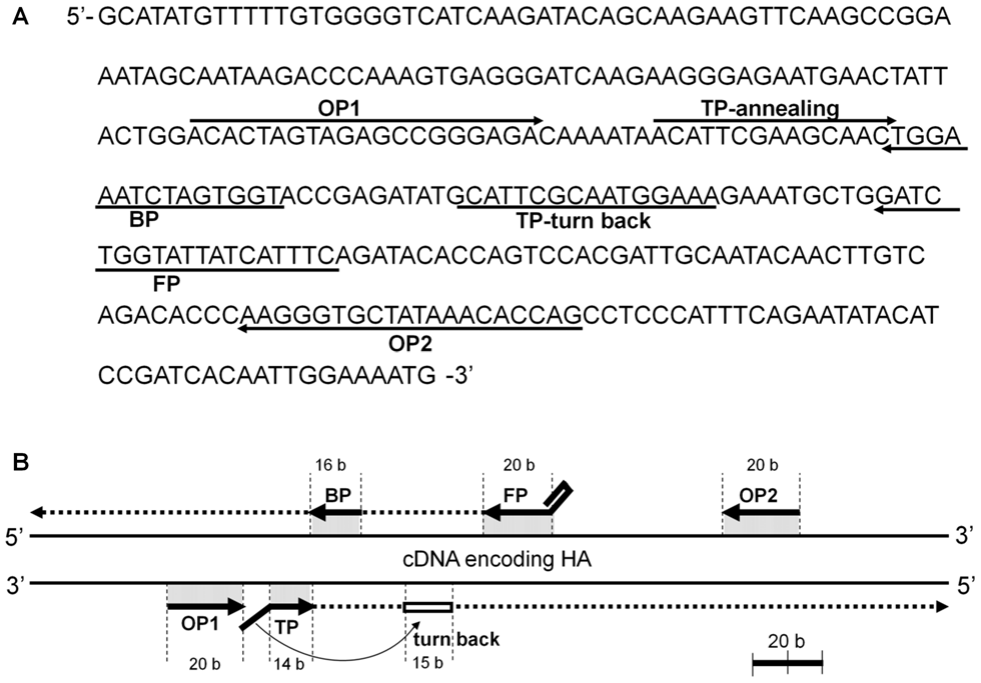
The sequence of nucleotides amplified by the SmartAmp reaction and primer annealing sites. **A:** cDNA encoding the partial sequence of the HA segment of the 2009 pdm influenza virus and primer annealing sites. **B:** Schematic illustration of annealing sites of the TP, FP, OP1, OP2, and BP primers. The size of each annealing site is numerically indicated as the number of base units (b).

The SmartAmp-based nucleotide amplification reaction requires five different primers: TP, FP, BP, OP1, and OP2 (see Materials and Methods). These primer candidates were selected on the basis of algorithms for free energy, probability of base-pairing, and product size range. After extensive screening with synthesized oligo-nucleotides as primer candidates, we have selected one optimal set of SmartAmp primers as shown in Table 1. The sequence of nucleotides amplified by the SmartAmp reaction and primer annealing sites are shown in Figures 2A and 2B, respectively. The genomic sequence located between the annealing sites of the TP and FP primers is the target region that is amplified by the SmartAmp reaction. In DNA amplification by SmartAmp, the very important initial step of copying the target sequence from the template DNA [16] is called an intermediate product generation step. The OP1 and OP2 primers were additionally used to accelerate the DNA strand-displacement process mediated by N-terminal-deleted *Aac* DNA polymerase.

**Table 1:** SmartAmp primers designed for detection of the 2009 pdm influenza A(H1N1) virus.

Primer	5′-DNA sequence-3′
TP	TTCCATTGCGAATGCACATTCGAAGCAAC
FP	GCATTCGCGAAATGATAATACCAGATCC
BP	ACCACTAGATTTCCAG
BP-Ex	ACCAC**Z**AGATTTCCAG
OP1	ACACTAGTAGAGCCGGGAGA
OP2	CTGGTGTTTATAGCACCCTT

TP: The turn back region is underlined.

FP: The folding region is underlined.

BP-Ex: The Exciton dye is covalently bound with thymidine that is marked with a bold “Z”.

After the determination of an optimal primer set, 25% of BP was replaced by Exciton dye-linked BP, namely, BP-Ex, that functioned as a “sequence-specific fluorescent primer” [19], [20]. In the BP-Ex primer, one thymidine was covalently linked with two thiazole orange molecules (Table 1). After hybridization to complementary sequences, the fluorescence of the BP-Ex primer provided sequence-specific signals for real-time monitoring of the DNA amplification reactions. We have examined the sensitivity of the primer set in the SmartAmp reaction by using various copy numbers of a plasmid DNA carrying an insert of the target sequence. The minimal level for detection was found to be 50 copies in the SmartAmp reaction mixture (25 µl) (Figure S1).

### Sensitivity and selectivity of the RT-SmartAmp assay

Since our aim was to develop the RT-SmartAmp assay method for detecting the HA segment of the 2009 pdm influenza A(H1N1) virus, the assay mixture contained 0.25 units of AMV reverse transcriptase (RT), as described in Materials and Methods. Figure S2 illustrates our strategy for the RT-SmartAmp assay reaction, where the first cDNA stand is synthesized from the viral RNA negative strand encoding the HA segment and two DNA intermediates are created in the subsequent SmartAmp reaction.

**Figure 3:**
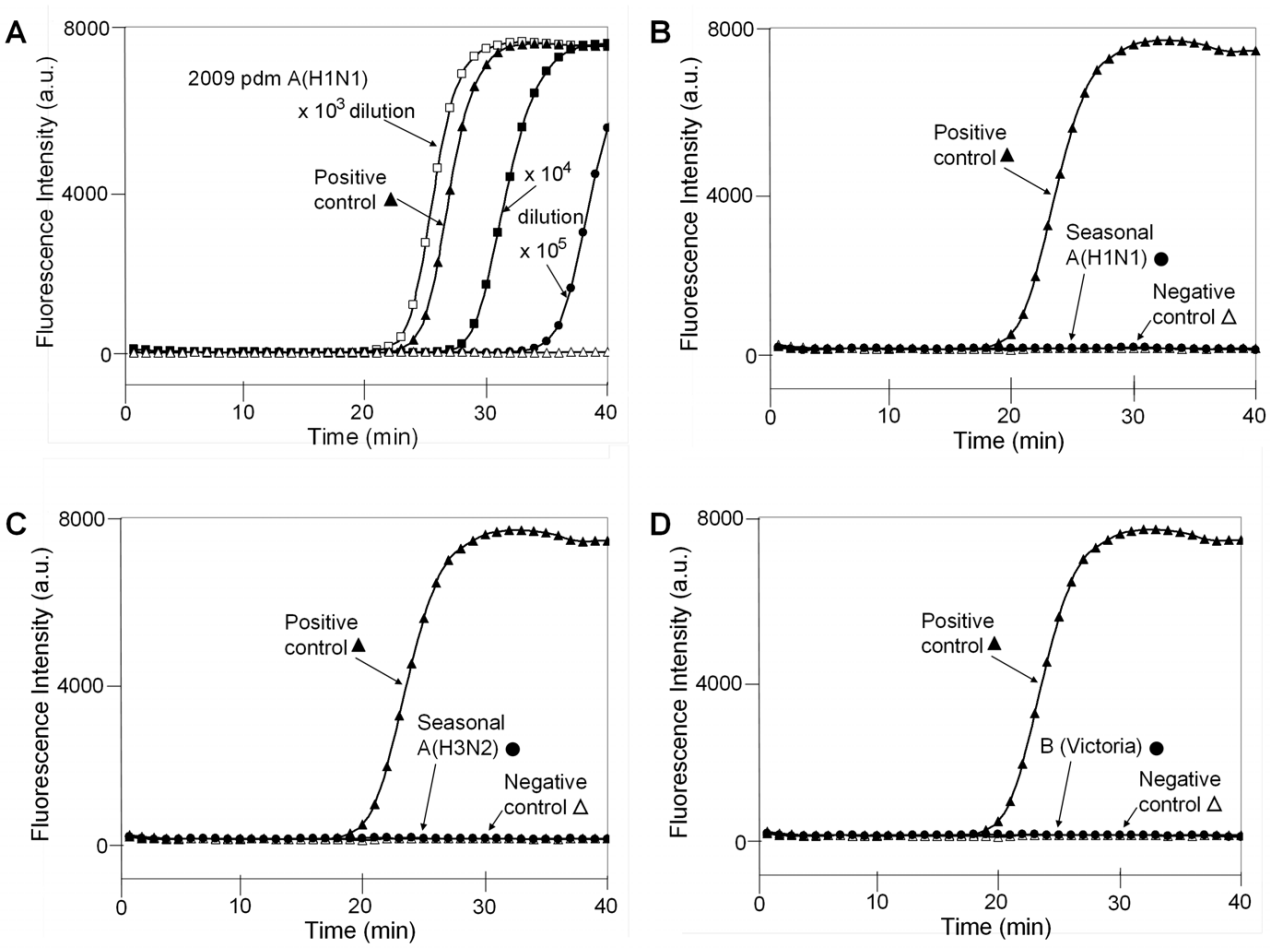
RT-SmartAmp detection of the 2009 pdm influenza A(H1N1) virus with different dilutions as well as the cross activity with seasonal A(H1N1), seasonal A(H3N2), and B(Victoria) viruses. Isolated and cultured influenza viruses, *i.e.*, 2009 pdm A(H1N1), seasonal A(H1N1), seasonal A(H3/N2), and seasonal B/Victoria, were prepared at a viral titer of 10^7^ pfu/ml. Each viral sample (10 µl), except for the 2009 pdm influenza A(H1N1) virus, was mixed with 90 µl of the pretreatment medium (5% SDS) to dissolve the viral membrane and to facilitate viral RNA extraction. A sample (15 µl) of the resulting medium was subjected to spin column chromatography, and the eluted solution (5 µl) was applied to the RT-SmartAmp reaction mixture. In the case of the 2009 pdm influenza A(H1N1) virus, the viral sample was diluted by 10^3^-, 10^4^-, or 10^5^-fold as indicated in the figure, and then processed in the same manner as described above. The RT-SmartAmp assay was performed as described in Materials and Methods.

Furthermore, we have established a simple pretreatment procedure, whereby we used a pretreatment medium containing 5% SDS to dissolve the viral membrane and to facilitate viral RNA extraction. SDS was thereafter removed by spin chromatography in a micro-tube column packed with Sephacryl S-400 HR gel-filtration resin. In combination with the simple pre-treatment procedure, we examined the sensitivity and selectivity of the RT-SmartAmp assay to the 2009 pdm influenza A(H1N1) virus. For this purpose, we used isolated and cultured influenza viruses, *i.e.*, 2009 pdm A(H1N1), seasonal A(H1N1), seasonal A(H3/N2), seasonal B (Victoria). Each of them had a viral titer of 10^7^ pfu/ml. Ten microliters from each viral sample was mixed with 90 µl of pretreatment medium (5% SDS). Subsequently, 15 µl of the suspension was subjected to spin column (Sephacryl S-400 HR) chromatography. Five microliters of the eluted solution was then applied to the reaction mixture of the RT-SmartAmp assay. Figure 3 demonstrates the reaction time courses, where the fluorescence of BP-Ex was continuously monitored. This RT-SmartAmp assay was specific to the 2009 pdm influenza A(H1N1) virus and showed no cross activity with seasonal A(H1N1), seasonal A(H3/N2), or seasonal B (Victoria). Even after a 10^5^-fold dilution, the 2009 pdm A(H1N1) virus could still be detected by the RT-SmartAmp assay with the same procedure (Figure 3).

We further tested the cross reactivity of the RT-SmartAmp assay with other infectious viruses and bacteria, as listed in Table S1, and confirmed that the RT-SmartAmp reaction was specific to the 2009 pdm influenza A(H1N1) virus. In this experiment, however, lysis of *Escherichia coli*, *Klebsiella pneumoniae*, and *Serratia marcescens* was not completely achieved by the pre-treatment (5% SDS). Therefore, these bacteria could not be subjected to the RT-SmartAmp assay.

### Clinical study to validate the RT-SmartAmp assay

In the present study, the RT-SmartAmp assay was clinically validated at three hospitals, *i.e.*, Chiba Prefectural Togane Hospital, Isumi Medical Center in Chiba, and the National Center for Global Health and Medicine in Tokyo [6]. About six months after the first case of infection with the 2009 pdm influenza virus was reported in Japan in May 2009, the influenza cases reported per sentinel peaked in the 48th epidemic week [6]. By that time, the 2009 pdm influenza A(H1N1) viruses had become the dominant strains [6]. From October 13, 2009, to January 6, 2010, during the peak phase of the pandemic in Japan, we collected a total of 255 samples from outpatients with influenza-like symptoms at these three hospitals and eleven clinics associated with Chiba Prefectural Togane Hospital.

Figure S3 depicts our scheme for the processing of nasopharyngeal swabs from patients and the detection of the 2009 pdm influenza A(H1N1) virus. Two swab samples were collected from each patient. One of them was used for the rapid influenza diagnostic tests, and the other sample was subjected to the RT-SmartAmp assay of the 2009 pdm influenza A(H1N1) virus. Both samples were then transferred to RIKEN Yokohama Institute for sequence analysis of the HA segment.

Before carrying out the 2009 pdm influenza A(H1N1) virus detection in clinical samples by the RT-SmartAmp assay, we defined the following criteria for our decision of positive or negative results. (1) Each assay must meet the two requirements of no amplification with the negative control and amplification with the positive control. (2) When the fluorescence intensity is higher than or equal to 3000 arbitrary units (a.u.) in an Mx3000P real-time PCR machine at 40 min of the RT-SmartAmp assay, the sample is regarded as positive for infection. (3) When the fluorescence intensity is lower than 3000 a.u. at 40 min of the RT-SmartAmp assay, the sample is regarded as negative for infection.

Among the 255 collected samples, 140 samples (54.9%) were detected by the RT-SmartAmp assay as positive in terms of infection with the 2009 pdm influenza A(H1N1) viruses. In contrast, by the rapid influenza diagnostic tests, 110 samples (43.1%) were detected as positive for influenza A infection. Table 2 summarizes the assay results obtained with all of the nasopharyngeal swabs from patients in this clinical study. There were 6 samples collected for this study that were scored as negative by the RT-SmartAmp assay, whereas the commercial rapid testing kit gave positive results for influenza A infection. To investigate this discrepancy, we performed quantitative RT-PCR (qRT-PCR) and sequence analysis for those 6 samples and found that each of them contained the 2009 pdm influenza A(H1N1) virus at under detectable levels, whereas sufficient levels of the 2009 pdm influenza A(H1N1) virus were detected in the samples collected for the rapid testing. It is likely that such differences in the virus levels occurred in the process of sampling, because we collected two separate nasopharyngeal swabs from each patient.

**Table 2:** Comparison of results detected by the rapid diagnosis kits and the RT-SmartAmp assay.

Rapid diagnosis kit	RT-SmartAmp assay	Number of paired samples	%	Remarks
Influenza A	2009 pdm A(H1N1)	104	40.8	2009 pdm A(H1N1) confirmed by sequence analysis
Influenza A	negative	6	2.4	Viruses under detectable level in RT-SmartAmp samples
negative	2009 pdm A(H1N1)	36	14.1	2009 pdm A(H1N1) confirmed by sequence analysis
negative	negative	109	42.7	Viruses under detectable level in both samples

As depicted in Figure S3, two swab samples were collected as one pair from each patient. Swab samples were separately used for the rapid influenza diagnostic tests and RT-SmartAmp assay. All the samples were transferred to RIKEN Yokohama Institute for sequence analysis and RT-Q-PCR detection.

### Clinical significance of the RT-SmartAmp method

A total of 132 patients were diagnosed as positive for infection with the 2009 pdm influenza A(H1N1) virus by the RT-SmartAmp assay. It should be kept in mind that, in some cases, multiple samples were collected from patients at different time points. In particular when the patients had very serious conditions or oseltamivir-resistance, multiple samples, *i.e.*, nasopharyngeal swabs and tracheal fluid samples, were collected. Therefore, the number (140) of samples was greater than the number (132) of patients. For the 2009 pdm influenza A(H1N1)-positive patients diagnosed by the RT-SmartAmp assay, we analyzed clinical information with respect to the time from fever onset until the sampling of nasopharyngeal swabs and summarized these data in Table 3. It is noteworthy that, in 72.8% of the infection-positive cases, the RT-SmartAmp assay could detect the 2009 pdm influenza A(H1N1) virus within 24 hours after the onset of fever. Moreover, in about 20% of the cases, the virus was positively detected by the RT-SmartAmp assay even less than 6 hours after the onset of fever (Table 3). These results suggest that the RT-SmartAmp assay is sensitive and clinically useful for detection of the 2009 pdm influenza A(H1N1) virus in early stages after the initial onset of flu symptoms.

**Table 3:** Time after the onset of fever and the number of patients who were diagnosed by the RT-SmartAmp assay as positive for infection with the 2009 pdm influenza A(H1N1) virus.

Time after onset of fever (hours)	Number of patients	%
0<Time≤6	26	19.7
6<Time≤12	15	11.4
12<Time≤18	33	25.0
18<Time≤24	22	16.7
24<Time	36	27.2

### Fatal case

**Figure 4:**
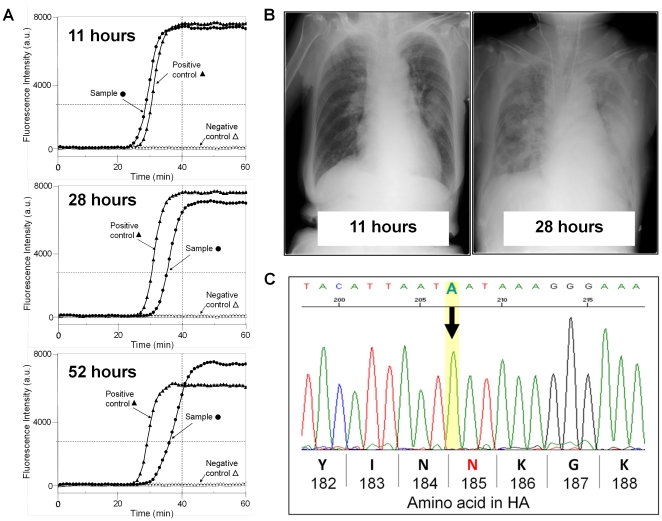
Detection of the 2009 pdm influenza A(H1N1) virus by RT-SmartAmp assay in the fatal case. **A:** Nasopharyngeal swab samples were collected at 11, 28, and 52 hours after the onset of fever from the patient who was transferred by ambulance to the National Center for Global Health and Medicine. The 2009 pdm influenza A(H1N1) virus was immediately detected by the RT-SmartAmp assay as described in Materials and Methods. **B:** Chest radiography of the patient was taken at 11 and 28 hours after the onset of fever. **C:** Partial sequence of the HA segment of the 2009 pdm influenza A(H1N1) virus was analyzed after extraction of viral genome RNA from the swab samples. An arrow indicates the mutation that caused an amino acid substitution at 185 from aspartate to asparagine (N) in the HA protein.

In the present clinical study, we encountered one fatal case, wherein a 72-year-old female had preexisting autoimmune hepatitis with liver cirrhosis. She received daily treatment with 5 mg of prednisolone and 50 mg of azathioprine. Flu-like symptoms had developed 11 hours before she was transferred by ambulance to the National Center for Global Health and Medicine (NCGM) in Tokyo. Upon admission, the patient was conscious and able to respond to the doctor's queries. At that time, the result of the rapid influenza diagnostic test was negative, whereas the RT-SmartAmp assay gave a positive result from her nasopharyngeal swab sample (Figure 4A). Therefore, the treatment with oseltamivir was started immediately. Nine hours later, however, her respiratory condition deteriorated, and she experienced a sudden drop in blood pressure. It was considered a diagnosis of severe influenza virus pneumonia complicated by septic shock. The patient was admitted to the intensive care unit (ICU) and promptly treated with broad-spectrum antibiotics, fluid resuscitation, norepinephrine, corticosteroids, and double dose oseltamivir. Figure 4B depicts the chest radiography of the patient, demonstrating a drastic progression of severe viral pneumonia, which is characterized by pathological lesions of diffuse alveolar damage (DAD). Both nasopharyngeal swab and tracheal fluid samples were taken from the patient and tested with a rapid influenza diagnostic kit and the RT-SmartAmp assay. The rapid influenza diagnostic kit again gave negative results with both swab and tracheal fluid samples. However, the RT-SmartAmp assay clearly detected the 2009 pdm virus in both samples (Figure 4A). It appeared that the 2009 pdm influenza A(H1N1) virus level was gradually decreasing over time, as shown in Figure 4A. In spite of intensive treatments, however, the patient expired 52 hours after the onset of fever.

Our sequence analyses revealed that the amino acid residue 185 in the HA protein of the 2009 pdm influenza A(H1N1) virus was asparagine (N), instead of aspartic acid (D) (Figure 4C), in both swab and tracheal fluid samples collected from the patient. HA-Asn185 is located in the Ca antigenic site [6], [21], being found in the HA protein of the reported sequences of the 1918 influenza viruses [22]–[23]. There was no mutation associated with oseltamivir-resistance in the NA segment in this patient (data not shown).

### Oseltamivir-resistance case

**Figure 5:**
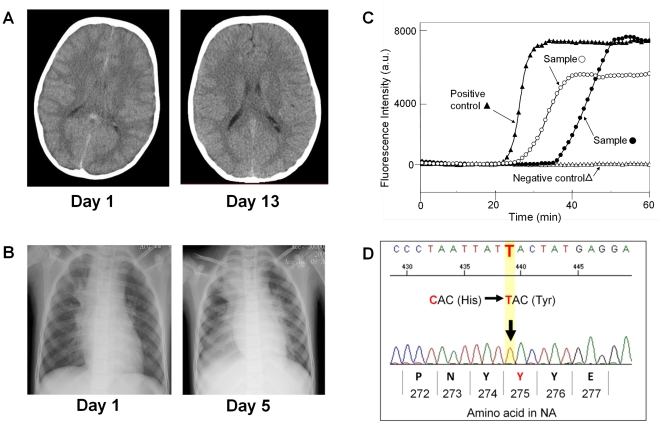
Detection of the 2009 pdm influenza A(H1N1) virus by the RT-SmartAmp assay in the oseltamivir-resistance case. **A:** Images of the head CT scanning taken on hospital day 1 (left) and day 13 (right). **B:** Chest radiography of the patient taken on hospital day 1 (left) and day 5 (right). **C:** The RT-SmartAmp assay with tracheal fluid (○) and nasopharyngeal swab (•) samples collected on hospital day 9. This figure depicts the time courses of the RT-SmartAmp assay reactions with those samples as well as with positive (▴) and negative (▵) controls. **D:** Partial sequence of the NA segment of the 2009 pdm influenza A(H1N1) virus was analyzed after extraction of viral genome RNA from the swab samples. An arrow indicates the mutation that caused an amino acid substitution at 275 from histidine (N) to tyrosine (Y) in the NA protein.

We encountered three oseltamivir-resistance cases in our clinical research. Here, we report a case of severe pneumonia and encephalopathy associated with oseltamivir-resistant 2009 pdm influenza A(H1N1) virus. A 6-year-old girl who had been vaccinated against 2009–10 seasonal influenza was transferred to NCGM in Tokyo with an 8-hour history of fever (39.9°C) and chills. Soon after admission, she rapidly became comatose with febrile convulsions. Nasopharyngeal swab samples were collected from the patient, and the rapid influenza diagnostic test showed infection positive with influenza A. A brain CT scan revealed diffuse and severe edema of the whole brain (Figure 5A). Moreover, chest radiography of the patient demonstrated slight infiltrates in both middle and lower lung fields and mild atelectasis of the right upper lung lobe (Figure 5B). Therefore, the patient was immediately treated with broad-spectrum antibiotics, high-dose corticosteroids, and oseltamivir (4 mg/kg/day) in the ICU. Nevertheless, 6 hours later, she fell into critical respiratory failure with a ratio of the partial pressure of arterial oxygen (PaO_2_) to the fraction of inspired oxygen (FIO_2_) of 123, and required mechanical ventilation. Despite the treatments, she produced more respiratory secretions, which were thick and sticky, and atelectasis of her lung progressed gradually. The PaO_2_/FIO_2_ ratio of the patient declined to 87 on hospital day 5, and bronchial toilet with bronchoscopy was required. Both nasopharyngeal swabs and tracheal fluid samples were tested with the rapid influenza diagnostic kit and the RT-SmartAmp assay, and the results were all positive (data not shown).

On hospital day 9, her respiratory condition gradually improved to a PaO_2_/FIO_2_ ratio of 207 with the treatments including repeated bronchoscopy for the purpose of bronchial toilet and respiratory physical therapy with discontinuance of skeletal muscle relaxant. Both nasopharyngeal swabs and tracheal fluid samples were collected and subjected to the rapid influenza diagnostic test and the RT-SmartAmp assay. The rapid influenza diagnostic kit gave negative results, whereas the RT-SmartAmp assay still detected the 2009 pdm influenza A(H1N1) virus in the tracheal fluid sample (Figure 5C). Therefore, the patient was treated with oseltamivir for 14 days. The patient was successfully weaned from mechanical ventilation on hospital day 13. A repeat brain CT scan showed alleviated edema in her whole brain (Figure 5A). While brain edema had to be carefully followed, the patient's condition gradually improved and her respiration was well stabilized. Finally, it took a total of 55 days until the patient recovered sufficiently to be discharged from the hospital.

Sequence analyses with both nasopharyngeal swabs and tracheal fluid samples from this patient revealed that the amino acid residue 275 in the NA protein of the 2009 pdm influenza A(H1N1) virus was tyrosine (Y), instead of histidine (H) (Figure 5D), providing evidence that this patient was infected with oseltamivir-resistant viruses. Furthermore, this clinical case has demonstrated that the RT-SmartAmp assay is sensitive for detecting the 2009 pdm influenza A(H1N1) virus not only in nasopharyngeal swabs but also in tracheal fluid samples.

## Discussion

### Clinical validation of SmartAmp-based detection of the 2009 pdm influenza A(H1N1) virus

Influenza A viruses belong to the family Orthomyxoviridae, containing a genome composed of eight segments (*i.e.*, PB1, PB2, PA, HA, NP, NA, M, and NS) of single-stranded negative-sense RNA [10]. The HA segment RNA encodes haemagglutinin protein, which is critical for viral binding to cellular receptors and fusion of the viral and endosomal membranes. We have selected the HA segment as a target for the RT-SmartAmp assay, since the segment gene represents the subtype-specific characteristics of influenza A viruses. The HA gene of the 2009 pdm influenza A(H1N1) viruses was reportedly derived from “classical swine H1N1” virus, which likely shares a common ancestor with the human H1N1 virus that caused the influenza pandemic in 1918, and whose descendant viruses continued to circulate in the human population with highly altered antigenicity of HA [9], [24], [25]. Studies on the worldwide evolution of the 2009 pdm influenza A(H1N1) viruses have shown that the 2009 pdm influenza A(H1N1) viruses appear to have a high mutation rate [26], [27]. In our recent study [6] as well as that of Shiino *et al.* [7], phylogenetic analyses carried out on Japanese isolates corroborated the circulation of four clusters, whereas most of the Japanese isolates were further divided into a total of 24 micro-clades [6].

While we selected the target region (Figure 1A) by considering the criteria of (*a*) low mutation rates and (*b*) high difference scores, we have investigated whether mutations occurred in the primer-annealing regions in the HA segment and whether such mutations could affect the RT-SmartAmp assay. As demonstrated in Figure S4, sequence analysis of the HA segment revealed that several mutations occurred in the primer-annealing regions in some swab samples. For the SmartAmp reaction, TP is the critically important driver of the amplification process [28]. In the present clinical study, two mutations were detected, one each in the TP-annealing and TP-turn back sites. In 14 samples, mutations were found in the OP1-annealing region, whereas one mutation was found in the OP2-annealing region in one sample (Figure S4). Nevertheless, the RT-SmartAmp assay was not greatly affected by those mutations in the HA segment.

The minimal virus level for recognition by the RT-SmartAmp assay was estimated to be 50 copies in the reaction mixture (25 µl). This value was comparable to that of qRT-PCR (the Real Time ready Influenza A(H1N1) Detection Kit from Roche Diagnosis GmbH). In this study, we could not directly compare the result of the RT-SmartAmp assay with that of the Human Influenza Virus Real-Time RT-PCR Detection and Characterization Panel (rRT-PCR Flu Panel) of the Centers for Disease Control and Prevention (CDC), since the rRT-PCR Flu Panel was not timely available when we started our clinical research. Nevertheless, as demonstrated in Table 3, in 72.8% of all the infection-positive cases, the RT-SmartAmp assay could detect the 2009 pdm influenza A(H1N1) virus within 24 hours after the onset of fever. Thus, the RT-SmartAmp assay is considered to provide a simple and practical tool to rapidly detect the 2009 pdm influenza A(H1N1) virus.

### The RT-SmartAmp assay as a point-of-care technology

Methods widely used for the definitive diagnosis and classification of influenza virus infections are mainly PCR-based. PCR-based diagnosis, however, is still relatively complex and expensive, since PCR requires thermocycling to mediate DNA melting, primer annealing, and polymerase-aided extension of DNA. More importantly, PCR-based techniques require *Taq* DNA polymerase, which is easily inhibited by impurities, and isolation of viral RNA is a prerequisite. Conversely, isothermal nucleic acid amplification technologies use a single reaction temperature, which translates into less complex and less expensive instrumentation. Indeed, the isothermal amplification technologies are rapidly growing to cover various applications, such as pathogen detection and SNP genotyping [29]–[32]. The SmartAmp method has been developed based on the concept that DNA amplification itself is the signal for the presence of a specific target sequence. Differing from the widely-used PCR, the SmartAmp reaction is an isothermal DNA amplification [16]–[18]. This is a great advantage for our rapid detection of influenza A viruses, since we can carry out both RT and isothermal DNA amplification reactions in one reaction tube. In the SmartAmp method, patient samples are processed by using the enzyme *Aac* polymerase. This enzyme is highly resistant to cellular contaminants.

In addition, we have most recently developed exciton-controlled hybridization-sensitive fluorescent primers [19], [20], named “Exciton Primers”, which significantly enhance the signal/noise ratio. Exciton Primers function as sequence-specific dyes. After hybridization to complementary sequences, the Exciton Primer provides a sequence-specific fluorescent signal for real-time monitoring of amplification reactions [22]. Exciton Primers show high signal strength with low background leading to a superior specificity and sensitivity compared to the commonly used SYBR® Green I [22]. Owing to their high signal/noise ratio, Exciton Primers enabled the visual end-point detection of 2009 pdm influenza A(H1N1) viruses by the RT-SmartAmp assay (data not shown).

### Comparison with other isothermal amplification methods

Recently, a reverse transcription-loop-mediated isothermal amplification (RT-LAMP) assay has been developed to detect the 2009 pdm influenza A(H1N1) virus [33]–[35]. In particular, a pocket-warmer RT-LAMP [35] may have great potential utility as an on-site diagnosis tool. LAMP is mainly used for pathogen detection with rapid and accurate detection [32], whereas SmartAmp is a particularly useful method for SNP and mutation detection [16], [18]. It is well known that the His-to-Tyr substitution at amino acid residue 275 confers the 2009 pdm influenza A(H1N1) virus oseltamivir-resistance, and this mutation was detected in patients infected with 2009 pdm influenza A(H1N1) viruses in our clinical study [6]. It has been reported that the oseltamivir-resistant 2009 pdm influenza A(H1N1) viruses were as pathogenic and transmittable as their drug-sensitive counterparts [14]. Since oseltamivir has been heavily used in pharmaceutical treatments in Japan, the oseltamivir-resistance rate is expected to greatly increase in the second pdm virus wave. Therefore, the next challenge for the RT-SmartAmp assay is to develop a kit for rapid detection of the mutation causing oseltamivir-resistance.

Furthermore, sample preparation is a bottleneck for nucleic acid amplification methods, including SmartAmp and LAMP as well as PCR-based clinical diagnostic applications. In the present study, we used the pretreatment medium containing 5% SDS to dissolve the viral membrane and to facilitate viral RNA extraction. Therefore, the SDS had to be removed from the sample by spin column chromatography. This step should be improved in future technology development. Sample preparation starting from clinical specimens, such as nasopharyngeal swabs, needs to be coupled with amplification and detection to achieve the final goal of point-of-care technologies.

### Concluding remarks

In conclusion, the present clinical study has verified the practical usefulness and high sensitivity of the RT-SmartAmp assay for detection of the 2009 pdm influenza A(H1N1) virus. Whilst the pandemic tapered off in 2011, outbreak and rapid spread of influenza virus infection is one of serious concerns in the North-East area of Japan. On March 11, 2011, extremely strong earthquakes and huge “Tsunami” waves attacked counties and provinces in that area. Approximately 20,000 people were killed by the natural disaster. Over 100,000 people had to evacuate, and most of the refugees are presently staying in temporary residences urgently constructed in local areas. It is critically important for those refugees to prevent the outbreak of any kind of infections diseases including influenza.

The possibility of human-to-human transmission of highly pathogenic avian influenza A(H5N1) viruses is becoming a fear for human health and society. Since the first human cases appeared in 1997 in Hong Kong, A(H5N1) viruses have been circulating among avian species and have spread throughout Asia, Europe, and Africa, with sporadic transmission to humans [36]–[38] and reports of nearly 60% mortality [39]. In this context, simple, cost-effective, and highly sensitive methods should be developed to detect influenza A(H5N1) viruses. As demonstrated in this study, the RT-SmartAmp assay would provide a practical tool to support rapid diagnosis of influenza virus infection and to prevent pandemic as well as endemic infection among humans.

## Materials and Methods

### Collection of influenza viral sequences and data analyses

Nucleotide sequences of the HA segment of 2009 pdm influenza A(H1N1) viruses were obtained from the NCBI Influenza Virus Resource database (http://www.ncbi.nlm.nih.gov/genomes/FLU/SwineFlu.html), and the HA sequences (longer than 1600 bases) were aligned by using the MUSCLE program [36]. The base composition, *i.e.*, P_i_(x) (i = A, T, G, or C), was obtained for each position (x) in the aligned nucleotide sequence of the HA segment. The consensus sequences were determined as the bases of the highest base compositions. The mutation rate at each consensus sequence position was calculated as the summation of the non-consensus base compositions. Example: mutation rate P(x) = P_A_(x)+P_T_(x)+P_G_(x), in the case where the consensus nucleotide is C at position (x).

The nucleotide sequences of the HA segment of seasonal H1N1 viruses reported since the year of 2004 were also obtained from the NCBI Influenza Virus Resource database and aligned in the same way as described above. The consensus sequences of the HA segment of 2009 pdm and seasonal H1N1 viruses were then aligned according to [40]. The difference between 2009 pandemic and previous seasonal H1N1 viruses was calculated at each position (x) in the nucleotide sequence of the HA segments according to the following the formula:
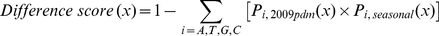
where x means the position in the HA nucleotide sequence, i = A, T, G, or C.

### Design of SmartAmp primer candidates

Based on the above-mentioned data analysis, one nucleotide region in the HA segment of the 2009 pdm influenza A(H1N1) virus was selected as a target candidate for SmartAmp primers. The criteria for selecting a target candidate were: (*a*) total mutation rates are low, and (*b*) total difference scores are high within a given partial nucleotide sequence. Candidates of SmartAmp primers were designed, *i.e.*, turnback primer (TP), boost primer (BP), forward primer (FP), and two outer primers (OP1 and OP2). The nucleotide sequence between the annealing sites of the TP and FP primers gives the actual target region to be amplified by the SmartAmp reaction. Primer candidates were selected based on algorithms [41] considering the free energy, probability of base-pairing, and product size range.

### Screening and optimization of SmartAmp primers

A variety of oligo-nucleotides were synthesized as primer candidates and screened in the standard SmartAmp reaction. Each reaction mixture (total volume of 25 µl) for the screening contained 1.82 µM each of FP and TP, 0.23 µM each of OP1 and OP2, 0.91 µM BP, 1.4 mM dNTPs, 20 mM Tris-HCl (pH 8.0), 10 mM KCl, 10 mM (NH_4_)_2_SO_4_, 8 mM MgSO_4_, 0.1% Tween® 20, 1/100,000 diluted original SYBR® Green I (TaKaRa BIO INC., Kyoto, Japan), 6 units of *Aac* DNA polymerase (K.K.DNAFORM, Yokohama, Japan), and about 10,000 copies of plasmid DNA with an insert of the target sequence. All experiments of the SmartAmp reaction were performed in an Mx3000P real-time PCR machine (Agilent Technologies, La Jolla, CA, USA) by maintaining the reaction temperature at 60°C and monitoring of changes in the fluorescence intensity with an FAM (band pass width of 492 nm–516 nm) filter.

### Synthesis of Exciton Primers

Exciton-controlled hybridization-sensitive fluorescent Primers tagged with thiazole orange, named “Exciton Primers”, were synthesized on solid support using standard phosphoramidite chemistry and purified by using HPLC, as described previously [19], [20]. After the primer set was determined, 25% of BP was replaced by BP Exciton Primer (BP-Ex).

### RNA synthesis by *in-vitro* transcription

RNA encoding the target sequence for the SmartAmp reaction was *in-vitro* synthesized by using the CUGA7® *in vitro* Transcription Kit (Nippongene, Japan) according to the manufacturer's protocol. Prior to the *in-vitro* transcription reaction, a template DNA fragment including a T7 promoter sequence was first created by PCR with SYBR® Premix Ex Taq™ (Takara Bio Inc., Japan), and the plasmid DNA carrying the target sequence. PCR primers (5′-CTAATACGACTCACTATAGGGCCATCTACTAGTGCTGACCA-3′ and 5′-CCCTTCA ATGAAACCGGCAA-3′) were designed to amplify the target sequence (positions 595–1048 in the HA segment). The PCR reaction was started with a DNA denaturation step (95°C for 5 min) and followed by a total of 35 cycles of thermocycling reactions (95°C for 30 sec, 60°C for 30 sec, 72°C for 30 sec) and a final extension reaction at 72°C for 3 min. The resulting PCR product was purified and used for the *in-vitro* transcription reaction. The quality of synthesized RNA was checked by using a Bioanalyzer with the total RNA nano kit (Agilent Technologies).

### Collection of swab samples from patients with flu symptoms in clinical research

The clinical research was conducted according to the Declaration of Helsinki Principles. Protocols for sample collection, storage, and SmartAmp-based detection of 2009 pdm influenza A(H1N1) viruses in swab samples obtained from patients were approved by the Institutional Review Boards at Chiba Prefectural Togane Hospital, Isumi Medical Center, and the National Center for Global Health and Medicine. Under written informed consent, nasopharyngeal swab samples were collected from patients with influenza-like symptoms at each of the hospitals and associated clinics. Transportation of those clinical samples to RIKEN was performed according to the guidelines provided by the National Institute of Infectious Diseases (Tokyo, Japan). Sequence analysis of the viral genome RNA was approved by the Research Ethical Committee at RIKEN Yokohama Institute.

### Pretreatment of swab samples

Pretreatment medium contained 5% sodium dodecyl sulfate (SDS). The swab sample from the patient was suspended in 300 µl of pretreatment medium with a vortex mixer. A gel-filtration spin column was prepared by swelling Sephacryl S-400 HR (GE Healthcare, Uppsala, Sweden) with Tris-EDTA buffer (TE buffer, pH 8.0) in a Micro Bio-Spin Chromatography Column (Bio-Lad Laboratories, Hercules, CA, USA). Then 15 µl of suspension was loaded onto the gel-filtration spin column, and was centrifuged at 1200× *g* for 1 min at room temperature. Five microliters of the eluted solution was applied to the reaction mixture.

### RT-SmartAmp assay

The RT-SmartAmp assay was performed on the reaction mixture (25 µl of final volume) containing 1.82 µM each of FP and TP, 0.23 µM each of OP1 and OP2, 0.68 µM BP, 0.23 µM BP-Ex, 1.4 mM dNTPs, 20 mM Tris-HCl (pH 8.0), 30 mM potassium acetate, 10 mM (NH_4_)_2_SO_4_, 8 mM MgSO_4_, 0.1% Tween® 20, 12 units of *Aac* DNA polymerase (K.K.DNAFORM, Yokohama, Japan), 0.25 units of AMV reverse transcriptase (RT) (Fermentus, Vilnius, Lithuania), into which 5 µl of the pretreated sample was mixed. To monitor the DNA amplification reaction proceeding at 60°C, the fluorescence of the BP-Ex primer was observed in an Mx3000P real-time PCR machine with the FAM filter (band pass width of 492 nm–516 nm).

### Real-time qRT-PCR for analysis of viral RNA copy number

The copy number of viral RNA extracted from swab samples was determined by quantitative RT-PCR (qRT-PCR) in a real-time PCR system LC480 (Roche, Basel, Switzerland), where the RealTime ready Influenza A(H1N1) Detection Set (Roche Diagnostics GmbH, Mannheim, Germany) and the RealTime ready RNA Virus Master (Roche Diagnostics) were used. The standard calibration curve for qRT-PCR was obtained by stepwise dilution of the *in-vitro* synthesized RNA with a known copy number.

### Analysis of viral genome sequence

Viral genome RNA (vRNA) was extracted from swab samples with the QIAamp Viral RNA Mini Kit (QIAGEN K.K., Tokyo, Japan) according to the manufacturer's instructions. A multisegment Reverse Transcription-PCR step was then performed on extracted vRNA by using universal influenza A primers under the conditions previously described by Zhou *et al.* [42], with the exception that we used 40 cycles, instead of 31, for the second cycle step [6]. Amplifications by PCR of the regions of interest were performed with Takara Ex Taq (TaKaRa BIO INC.) by using primers flanked with the T7 promoter for the forward primer and the SP6 promoter for the reverse primer. Two PCR primer sets were designed for the HA segment (positions 392 to 851 with 5′-taatacgactcactatagggGATTATGAGGAGCTAAGAGA-3′ and 5′-atttaggtgacactatagaaGATCCAGCATTTCTTTCCAT-3′, and positions 792 to 1251 with 5′-taatacgactcactatagggACTGGACACTAGTAGAGCCG-3′ and 5′-atttaggtgacactatagaaCTCTTTACCTACTGCTGTGA-3′) and one primer set for the NA segment (positions 417 to 976 with 5′- taatacgactcactatagggCCTTGGAATGCAGAACCTTC-3′ and 5′-atttaggtgacactatagaaGATTGTCTCCGAAAATCCCA-3′). Samples were then treated with ExoSAP-IT (GE Healthcare, Tokyo) and sequenced. The resulting PCR products were subjected to direct sequencing with a capillary 3730*xl* DNA Analyzer sequencer (Applied Biosystems, Tokyo, Japan) according to the manufacturer's protocol, as described previously [6].

## Supporting Information

Figure S1 **Time courses of the SmartAmp reaction during detection of various copy numbers of cDNA encoding the target sequence in the HA segment of the 2009 pdm influenza A(H1N1) virus.** The reaction mixture contained 1.82 µM each of FP and TP, 0.23 µM each of OP1 and OP2, 0.68 µM BP, 0.23 µM BP-Ex, 1.4 mM dNTPs, 20 mM Tris-HCl (pH 8.0), 10 mM KCl, 10 mM (NH_4_)_2_SO_4_, 8 mM MgSO_4_, 0.1% Tween® 20, 6 units of *Aac* DNA polymerase, and plasmid DNA with an insert of the target sequence of the HA segment. The copy number of the target sequence was 0, 50, 500, or 5,000 in the reaction mixture (25 µl of volume). The SmartAmp reaction was observed in an Mx3000P real-time PCR machine, where the reaction temperature was maintained at 60°C and the fluorescence of the BP-Ex primer was monitored over time.
(TIF)

Figure S2 **Schematic illustration of RT-SmartAmp reaction.** **A:** Formation of the first cDNA strand from the viral RNA negative strand encoding the HA segment as well as the subsequent steps of DNA polymerase reaction involved in the SmartAmp reaction with TP, FP, OP1, and OP2 primers. **B:** Creation of two DNA intermediate products derived from the SmartAmp reaction.
(TIF)

Figure S3 **Schematic illustration of sample processing for the rapid diagnosis test using immunochromatography kits and the SmartAmp assay-based detection of the 2009 pdm influenza A(H1N1) virus.** Two swab samples were collected from each patient to perform both the rapid influenza diagnostic test and the RT-SmartAmp assay to detect the 2009 pdm influenza A(H1N1) virus. Those detection methods required different pre-treatment procedures. vRNA was extracted from those pre-treatment media and subjected to multi-segment RT-PCR and sequence analysis, as described in Materials and Methods.
(TIF)

Figure S4 **Mutations found in the annealing sites of SmartAmp primers in the HA segment.** A total of 140 samples (RT-SmartAmp assay positive) were subjected to multi-segment RT-PCR and sequence analysis, as described in Materials and Methods. Red letters indicate the mutations found in the annealing sites of the SmartAmp primers in the HA segment. The number of samples for each mutation is given in the inset table.
(TIF)

Table S1 **Cross reactivity of the SmartAmp primers to various pathogens.** The cross reactivity was tested by using three different lots of the RT-SmartAmp primer set. ND, not determined.
(DOC)
