# Supplementary figures and images for: One-Step Detection of the 2009 Pandemic Influenza A(H1N1) Virus by the RT-SmartAmp Assay and Its Clinical Validation
Source: PLoS One. 2012 Jan 25;7(1):e30236. doi: 10.1371/journal.pone.0030236 (PMC3266250; doi:10.1371/journal.pone.0030236)

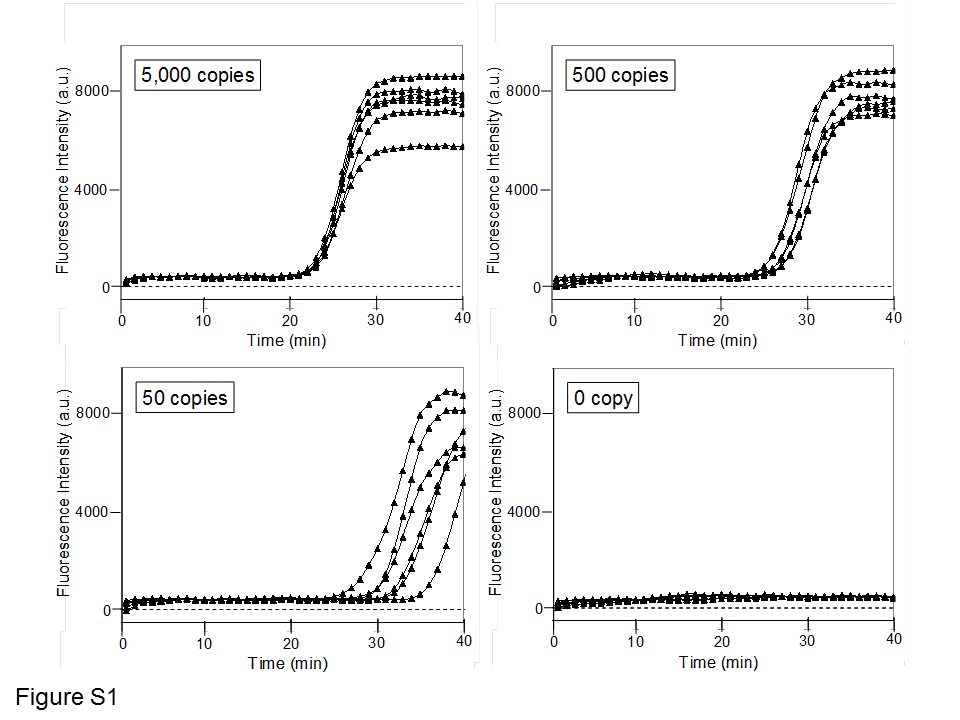

Supplement: Figure S1 — Time courses of the SmartAmp reaction during detection of various copy numbers of cDNA encoding the target sequence in the HA segment of the 2009 pdm influenza A(H1N1) virus. The reaction mixture contained 1.82 µM each of FP and TP, 0.23 µM each of OP1 and OP2, 0.68 µM BP, 0.23 µM BP-Ex, 1.4 mM dNTPs, 20 mM Tris-HCl (pH 8.0), 10 mM KCl, 10 mM (NH4)2SO4, 8 mM MgSO4, 0.1% Tween® 20, 6 units of Aac DNA polymerase, and plasmid DNA with an insert of the target sequence of the HA segment. The copy number of the target sequence was 0, 50, 500, or 5,000 in the reaction mixture (25 µl of volume). The SmartAmp reaction was observed in an Mx3000P real-time PCR machine, where the reaction temperature was maintained at 60°C and the fluorescence of the BP-Ex primer was monitored over time. (TIF) [file pone.0030236.s001.tif]

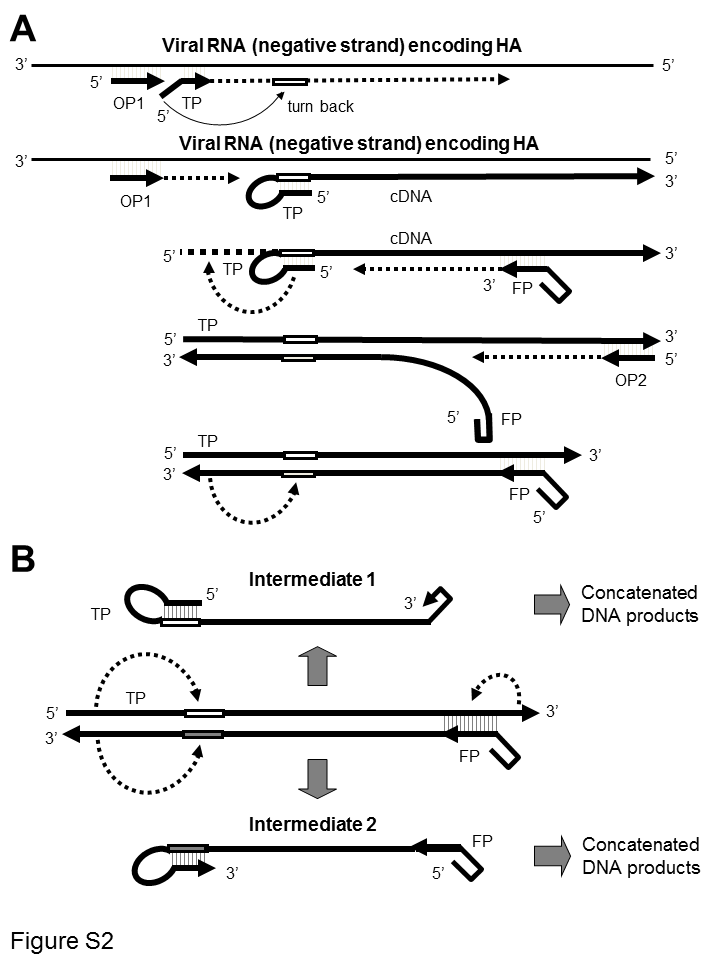

Supplement: Figure S2 — Schematic illustration of RT-SmartAmp reaction. A: Formation of the first cDNA strand from the viral RNA negative strand encoding the HA segment as well as the subsequent steps of DNA polymerase reaction involved in the SmartAmp reaction with TP, FP, OP1, and OP2 primers. B: Creation of two DNA intermediate products derived from the SmartAmp reaction. (TIF) [file pone.0030236.s002.tif]

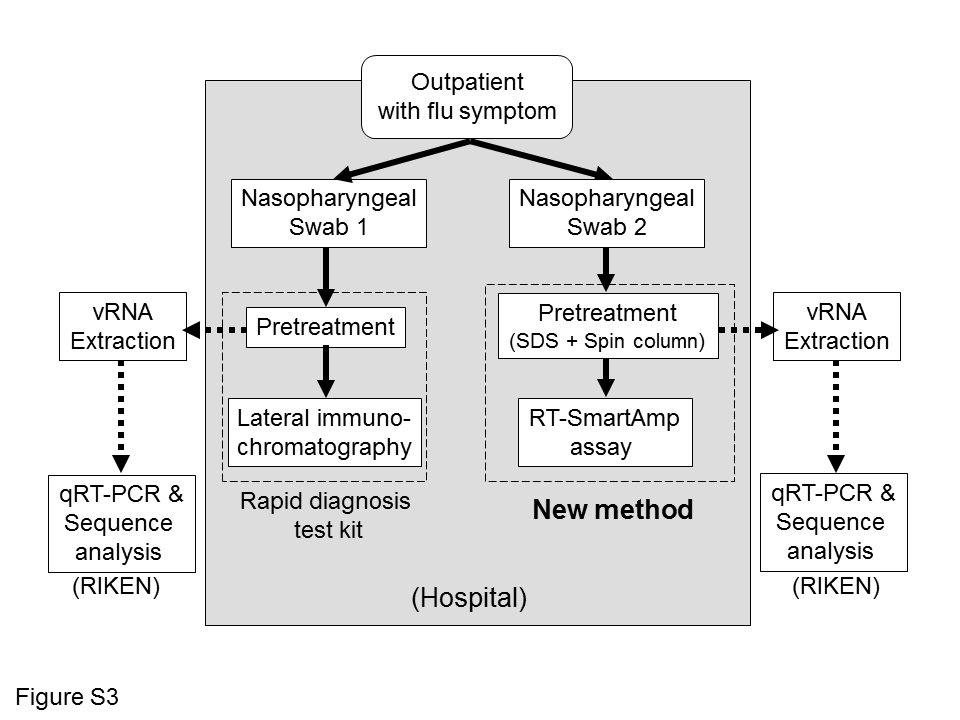

Supplement: Figure S3 — Schematic illustration of sample processing for the rapid diagnosis test using immunochromatography kits and the SmartAmp assay-based detection of the 2009 pdm influenza A(H1N1) virus. Two swab samples were collected from each patient to perform both the rapid influenza diagnostic test and the RT-SmartAmp assay to detect the 2009 pdm influenza A(H1N1) virus. Those detection methods required different pre-treatment procedures. vRNA was extracted from those pre-treatment media and subjected to multi-segment RT-PCR and sequence analysis, as described in Materials and Methods. (TIF) [file pone.0030236.s003.tif]

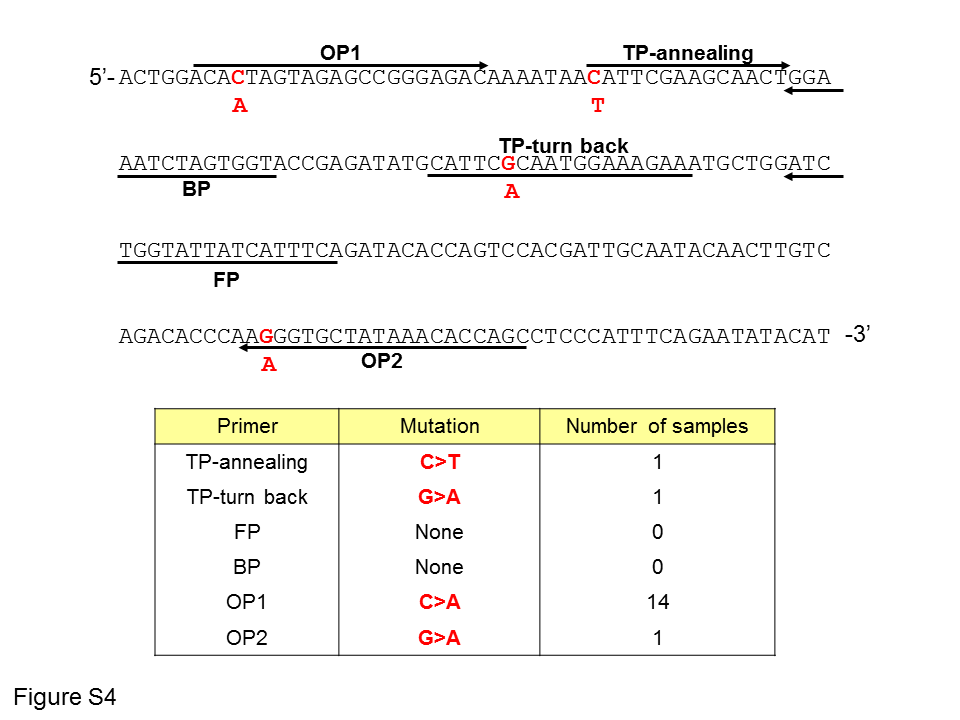

Supplement: Figure S4 — Mutations found in the annealing sites of SmartAmp primers in the HA segment. A total of 140 samples (RT-SmartAmp assay positive) were subjected to multi-segment RT-PCR and sequence analysis, as described in Materials and Methods. Red letters indicate the mutations found in the annealing sites of the SmartAmp primers in the HA segment. The number of samples for each mutation is given in the inset table. (TIF) [file pone.0030236.s004.tif]
