# Supplementary material for: One-Step Detection of the 2009 Pandemic Influenza A(H1N1) Virus by the RT-SmartAmp Assay and Its Clinical Validation
Source: PLoS One. 2012 Jan 25;7(1):e30236. doi: 10.1371/journal.pone.0030236 (PMC3266250; doi:10.1371/journal.pone.0030236)
Supplement: Table S1 — Cross reactivity of the SmartAmp primers to various pathogens. The cross reactivity was tested by using three different lots of the RT-SmartAmp primer set. ND, not determined. (DOC) [file pone.0030236.s005.doc]

**Supporting Information**

**Table S1.** Cross reactivity of the SmartAmp primers to various pathogens

| Pathogen | Type | SmartAmp result |
| --- | --- | --- |
| Viruses | Influenza 2009 pdm Type A (H1) | Positive (+ + +) |
|  | Influenza virus Type A (H1) | Negative (- - -) |
|  | Influenza virus Type A (H3) | Negative (- - -) |
|  | Influenza virus Type B | Negative (- - -) |
|  | Adenovirus Type 1 | Negative (- - -) |
|  | Adenovirus Type 2 | Negative (- - -) |
|  | Adenovirus Type 3 | Negative (- - -) |
|  | Adenovirus Type 4 | Negative (- - -) |
|  | Adenovirus Type 5 | Negative (- - -) |
|  | Adenovirus Type 6 | Negative (- - -) |
|  | Adenovirus Type 7 | Negative (- - -) |
|  | Adenovirus Type 8 | Negative (- - -) |
|  | Adenovirus Type 11 | Negative (- - -) |
|  | Adenovirus Type 19 | Negative (- - -) |
|  | Adenovirus Type 37 | Negative (- - -) |
|  | Coxsackievirus Type A2 | Negative (- - -) |
|  | Coxsackievirus Type A4 | Negative (- - -) |
|  | Coxsackievirus Type A5 | Negative (- - -) |
|  | Coxsackievirus Type A6 | Negative (- - -) |
|  | Coxsackievirus Type A8 | Negative (- - -) |
|  | Coxsackievirus Type A9 | Negative (- - -) |
|  | Coxsackievirus Type A10 | Negative (- - -) |
|  | Coxsackievirus Type A12 | Negative (- - -) |
|  | Coxsackievirus Type A14 | Negative (- - -) |
|  | Coxsackievirus Type A16 | Negative (- - -) |
|  | Coxsackievirus Type B1 | Negative (- - -) |
|  | Coxsackievirus Type B2 | Negative (- - -) |
|  | Coxsackievirus Type B3 | Negative (- - -) |
|  | Coxsackievirus Type B4 | Negative (- - -) |
|  | Coxsackievirus Type B5 | Negative (- - -) |
|  | Echovirus Type 3 | Negative (- - -) |
|  | Echovirus Type 6 | Negative (- - -) |
|  | Echovirus Type 9 | Negative (- - -) |
|  | Echovirus Type 11 | Negative (- - -) |
|  | Echovirus Type 13 | Negative (- - -) |
|  | Echovirus Type 14 | Negative (- - -) |
|  | Echovirus Type 18 | Negative (- - -) |
|  | Echovirus Type 30 | Negative (- - -) |
|  | Enterovirus Type 71 | Negative (- - -) |
|  | Parecho virus Type 1 | Negative (- - -) |
|  | Rhino virus | Negative (- - -) |
|  | Parainfluenza virus Type 3 | Negative (- - -) |
|  | Mumps virus | Negative (- - -) |
|  | Herpes Simplex virus Type 1 | Negative (- - -) |
|  | Parechovirus Type 3 | Negative (- - -) |
| Bacteria | Bordetella pertussis | Negative (- - -) |
|  | Enterococcus faecalis | Negative (- - -) |
|  | Enterococcus gallinarum | Negative (- - -) |
|  | Escherichia coli | ND |
|  | Haemophilus influenzae | Negative (- - -) |
|  | Klebsiella pneumoniae | ND |
|  | Legionella pneumophila | Negative (- - -) |
|  | Moraxella catarrhalis | Negative (- - -) |
|  | Mycoplasma pneumoniae | Negative (- - -) |
|  | Neisseria meningitidis | Negative (- - -) |
|  | Proteus mirabilis | Negative (- - -) |
|  | Pseudomonas aeruginosa | Negative (- - -) |
|  | Serratia marcescens | ND |
|  | Staphylococcus aureus | Negative (- - -) |
|  | Staphylococcus epidermidis | Negative (- - -) |
|  | Streptococcus group A | Negative (- - -) |
|  | Streptococcus group B | Negative (- - -) |
|  | Streptococcus group C | Negative (- - -) |
|  | Streptococcus group G | Negative (- - -) |
|  | Streptococcus mutans | Negative (- - -) |
|  | Streptococcus pneumoniae | Negative (- - -) |
|  | Corynebacterium diphtheriae | Negative (- - -) |

ND, not determined.

The cross reactivity was tested by using three different lots of the RT-SmartAmp primer set.
